# From Diagnosis to Treatment of Lung Cancer: An Update in “Cancers” in 2021

**DOI:** 10.3390/cancers14225639

**Published:** 2022-11-17

**Authors:** Francesco Petrella

**Affiliations:** 1 Department of Thoracic Surgery, IRCCS European Institute of Oncology, 435-20141 Milan, Italy; francesco.petrella@ieo.it or francesco.petrella@unimi.it; Tel.: +39-02-5748-9362; Fax: +39-02-9437-9218; 2 Department of Oncology and Hemato-Oncology, University of Milan, 435-20141 Milan, Italy

After its successful launch in January 2021 by *Cancers*, the topic collection “Diagnosis and Treatment of Primary and Secondary Lung Cancers” experienced a productive first full year. In 2021, six cutting-edge papers were published under this topic collection. Diagnostic, therapeutic, and prognostic articles have been published, focusing on early-stage, locally advanced, and metastatic stages of cancer. I had the pleasure of co-writing a paper about the prognostic role of the hemoglobin/red cell distribution width ratio in resected lung adenocarcinoma; in this paper we observed that the ratio of hemoglobin-to-red cell distribution width—which has been described as an effective prognostic factor in several types of cancer—is an effective prognostic factor of disease-free survival in resected-lung-adenocarcinoma patients, together with the level of pathologic node involvement [1].

Shalata et al. reviewed the literature about the modifications and advancements of treatments for non-small cell lung cancer (NSCLC), focusing on adjuvant therapy with tyrosine kinase inhibitors (TKI) administered to NSCLC patients after radical resection, harboring a mutated epidermal growth factor receptor [2].

Mielgo-Rubio et al. reviewed all therapeutic approaches to stage III-N2 NSCLC, analyzing both completed and ongoing studies evaluating the addition of immunotherapy with or without chemotherapy and/or radiotherapy [3].

With regard to the diagnostic approach to lung cancer, Ito et al. reported their experience in patients suffering from interstitial lung disease with lung cancer within or near fibrotic lesions. They evaluated the yield for peripheral pulmonary lesions (PPLs) using endobronchial ultrasonography with a guide sheath transbronchial biopsy (EBUS-GS TBB) according to the proximity of PPLs to fibrotic lesions and determined the factors affecting the yield for PPLs. They observed that peripheral pulmonary lesions that do no overlap and a probe position within the lesion were significant factors affecting the diagnostic yield. The positional relation of lesions to fibrotic lesions and the probe position were important factors affecting successful diagnosis via endobronchial ultrasonography with a guide sheath transbronchial biopsy in these patients [4]. The bronchoscopic approach—both via flexible and rigid bronchoscopy—still remains the gold standard approach for lung diagnosis, particularly for centrally located nodules [5].

Lang et al. explored the clinical impact of sex differences in two group of patients receiving immune-checkpoint inhibitor (ICI) monotherapy or an ICI chemotherapy (ICI—CHT) combination.

They observed no significant difference in the outcomes between men and women treated with either therapeutic regimen. On the other hand, known predictive factors for ICI response—such as the expression of programmed-death ligand 1 (PD-L1) on tumor cells or patient performance status—had significant implications for men but not for women [6].

In conclusion, we had an initial overview of the most important aspects of lung cancer diagnosis, therapy, and prognosis, and during 2022, further high-quality papers have been submitted to the topical issue. We will further focus on rare primary pulmonary tumors, oligometastatic disease, and major complications after extended resection, thus further expanding our clinical overview on the diagnosis and treatment of primary and secondary lung cancers [7,8,9].
